# Veratrum Viride

**Published:** 1857-06

**Authors:** D. L. M’Gugin

**Affiliations:** Professor Physiology, Pathology, and Clinical Medicine, Iowa University


### VERATRUM VIRIDE.

REPORT OF CASES BY D. L. M'GUGIN, A. M. M. D.

Professor Physiology, Pathology, and Clinical Medicine, Iowa University.

The caution, so often repeated, that the practitioner should be guarded lest he fall a victim to that disease, which, at this day when insanity is made a plea of excuse for every form of moral obliquity, I am warranted in denominating "*hobby mania*," I would say that the caution is a wise one, and yet we may err in the opposite extreme. There is certainly a conservative line of demarkation between pliant credulity and medical bigotry and which, provided that it can be found, we are called upon by every consideration of duty to pursue.

The tendency to the adoption of preferences, imbibing prejudices, and of being blinded by partialities too often formed and constructed upon flimsey bases and specious appearances, is now so common that it is no rare spectacle to see a professional brother, with hurried steps and staring eyes *nolens volens* in the wake of a beguiling and deceptive *ignis fatuus*. The danger lies in first appearances, should these be rendered bright by a phosphorescent tint or two. Once astray they, like to the lost child, become confused, bewildered, and befogged, in a condition easily beguiled by delusions which under other circumstances characterized by calmness of mind and deliberative judgment, would have been promptly abandoned. If they are astray however in the first step, every one taken afterwards is wrong, conclusions thus imperfectly drawn are adopted as true, and the after history of their observations is not directed to the discovery of the truth or falsity of the evidence producing their first conviction, but adhering to the fidelity of first impressions, they overlook every circumstance which might tend to weaken their precipitate first-belief, pass all others by unheeded and unexamined, and seize upon minor facts as they hurriedly pass, simply because they indicate, however feebly, the object of their pursuit.

Not only so but the evidences, against their favorite belief, are tortured and twisted to suit their preconceived notions. Such is the condition of all *hobby riders* who urge their "ponies" beyond their speed and strength, and while the ears of their "nags" are in advance, they slide into the pleasing delusion that they are rapidly *onward*, whether the legs are capable of locomotion or the animals have lost their caudal appendage after the manner of old "Meg" in "Tam O'Shanter," are matters of small consequence.

Experience has so often verified this fact, when remedies have been over vaunted and individuals beguiled, that I hesitated in the use of the veratrum, believing that its therapeautic powers were overrated and an unexceptionable and *unfailing* character attributed to its use. All those who wrote concerning it—Dr. Norwood, *et. al.*, betrayed so much confidence,—so much passionate zeal in its favor, that I really began to rank it among the great and numerous family of *humbugs*. It is stated that "*that* which proves too much, just proves nothing at all," and this is an uncontroverted law of logic. A witness who testifies to more than the truth suffers the whole of his testimony to be ignored, the more especially, if he manifests much warmth in the relation. When I found that quinine was even less certain in its action, and opium a secondary remedy in importance when compared with the transcendant powers of the *Veratrum Viride*, I thought I saw in all this ardent—indeed I may almost say, vehement manner displayed in its behalf, a *well-groomed hobby*, bitted and caparisoned, ready for the *spur* and *whip*, but being an over-cautious rider, I was indisposed to try its bottom or speed.

The course which I decided upon, in this day, when a long experience is equivalent to *old fogyism* no matter even whether or not we have seen every new planet in the medical firmament during our long professional career,—this course I say would not be regarded as altogether *progressive*.

In this conclusion I might differ, with all due deference to juvenile "*America*" however, for although not always lenient and mild, experience is nevertheless a good master, and one which authorises me to judge for myself in the premises.

Dismissing these reflections, into the utterance of which I have been imperceptibly drawn too far, I feel myself in candor called upon to give my small experience with this remedy, for the benefit of those who may not have used it, and although the cases are few in number, yet their character, and the results of the treatment, justify the propriety, and indeed impose the duty, of making a report of them. In making out these cases I will not trouble the reader with particulars as to all the symptoms, but will very briefly refer to the facts in connexion with the use of the remedy.

In January last, I was required to prescribe for a little girl of nervous temperament, and laboring under what I believed to be lobular pneumonitis of the right lung. Used calomel. and tart antim. for several days with no lessening of the hearts action. Ventured upon the veratrum in two drop doses in *syrrup scillae simp.* Pulse still the same for 12 hours, the tincture was increased to 4 drops every 3 hours. In thirty-six hours the pulse had sunk from 140 to less than 100, the expectoration increased, the cough less distressing, and the skin moist. The tincture was continued a few days, during which the pulse was controlled by it and the patient rapidly convalesced.

The next case was that of an Irish girl, of 14 years, stout, vigorous and active, laboring under double pneumonia. Pulse 130. Commenced with 4 drops and increased to 6. In 12 hours the pulse came down to 86. The usual therapeutic effect had been induced, namely, irritation of the stomach with vomiting, the expectoration copious and free from blood globules, or the rust as it is termed, perspiration copious, skin warm, breathing less, all physiological symptoms improved, and the physical signs less manifest. The tincture was not given during one night, and toward morning the pulse became frequent again and quick; but a recurrence to the remedy had its former and desired effect. I dismissed her in six days after the attack rapidly convalescing.

The third case was that of a young man with strong proclivity to pulmonary disturbance and had suffered almost to "the death" with pneumonitis about three years since. It was fortunately single and confined to the right lung. The last attack from which he is slowly recovering was one of unusual severity, in the same lung, and threatened a fatal termination. So great was the inflammatory engorgement of the first stage that his face was not only livid but rather cyanotic. The respiration was hurried and laborious, pulse 130, skin dry and parched, sputa thick, tenaceous, and adhering to the vessel, stomach irritable, the dull sound upon percussion on the right side. It may not be a digression here to remark that, in all my experience in the early stage of pneumonitis, which is one of engorgement, the sound emitted by percussion is more or less dull, approximating to, though not actually, a flat sound. I mention this fact, in this connexion, because Skoda maintains that the resonance is unattended in the congestive first stage. unaltered

Auscultation gave the first sign of crepitant rale. Such were the leading physiological symptoms, and such the prominent physical signs, and I have been thus particular, because the experienced in this disease will see that the case was one of a very serious disturbance.

Calomel, combined with tart. antimony in small and repeated doses, was given with stimulating embrocations over the right chest, and his bowels moved by a mild cathartic, after which I ordered Tinct. Verat. Viridi in four drop doses with as many of syr. scilla. simp. every two hours—to be increased until nausea and vomiting should take place. In 12 hours the pulse came down to 80 full and soft, the skin covered with a free perspiration, expectoration easy and abundant, respiration 20, the face pale, the pain in the right side in the region of the lower lobe, which I had forgotten to say indicated inflammation of the pulmonary pleura, was relieved, in fine, all the symptoms and signs were in favor of my patient. The *Tinct. Verat. Vir.* was sometimes suspended when the unfavorable symptoms would increase, but soon after recurring to its use, its effects were immediately apparent. Owing to circumstances, not under my control, and certainly not in accordance with my wirhes, it was twice suspended after its good effects were first made apparent when there would be a return of unpleasant symptoms, but upon its exhibition again, the undesired phenomena would subside. It was however persisted in until the safety of the patient, by rapid and certain convalescence, warranted its discontinuance.

Four facts presented themselves in the progress of this case, namely: 1st. The tartarised antimony failed as a sedative. 2d. The Tinct. Verat. Vir. reduced the pulse in 12 hours from 110 to 80. 3d. Its suspension was followed by an increase of the symptoms,—and 4th, upon recurring to it, the results were always desirable and such as were first produced when first given. I might add to the above two other facts which were more or less made manifest in the use of this remedy in the cases above recited, and these were the manifest diaphoretic and expectorant effects of this remedy. This may have been observed by others, but I do not remember to have seen these therapeutic powers ascribed to its use by any of the writers upon the virtues of this medicinal agent. In one case I thougbt it proved a diuretic, but of this I am not so well assured.

The fourth case presented itself after the report of the previous cases had been made up. In this case we had a constitution very much shattered, and the natural vigor much impaired by previous disease. It proved to be pneumonitis in the first stage. After the attack, he had been packed in a blanket wrung out of cold water. The pulse was 110 and quick in its action. The face was purple and the respiration 24. The lower left lobe was engorged, giving the usual physical signs, and the right lower lobe was bridled doubtless by an adhering band between the pulmonary and costal pleura, so that the left lung was deficient in its functional capacity. The tongue was loaded and the secretions defective.

In view of his slender vital forces, I pondered upon the propriety of the exhibition of the *Veratrum,* but finally decided to do so as it was very important that the heart's action should be diminished, in order to lessen the amount of blood sent to the lungs. But caution governed the procedure; and but four drops were given in as many hours. As the pulse had not acknowledged its influence in 24 hours, the dose was increased to 5 drops and the interval only 3, instead of 4 hours. The second dose however, produced some nausea, accompanied with an effort to vomit, when the pulse fell down to 80. There was copious perspiration with warm skin, the breathing 16, the expectoration free and for the first time rusty, the bowels were freely moved, the urine copious and nearer the normal color, and there was an entire freedom from pain.

These symptoms had been so suddenly induced that it gave rise to alarm on the part of the patient and some of his friends, when I was forthwith summoned, and found his condition to be as represented above, which, of course, was most gratifying to my feelings.

Since then he has been steadily improving, the physical signs vanishing, and the other symptoms very encouraging, so much so, that at the present writing, he is out of his bed, free from cough and pain, and his pulse only 86 soft, and more full. But the tincture is continued in less doses, and with longer intervals.

After carefully watching the effects of this remedy, I am convinced that as an arterial sedative we have no superior, and that it fills a void in the list of our therapeautic agents which we have felt, and from the want of such agent we have all along labored and struggled. If it should prove, upon further trial, to be as potent in the control of the hearts action as in the few cases in which I have tried it, I will then look far back in the past and bring up to memory numerous cases in which I might have been able to have averted results which, with the ordinary remedies, could not be turned aside. The reminiscence would be painful and mortifying, and the more so, because of the length of time during which I was deprived of the benefits of a remedy so valuable and efficient. My opinion is, that, although it is a remedy which must be exhibited with care, judgment and circumspection, yet it may prove to be as certain in its own peculiar efficacy and powers as quinine, upon the powers of which we rely with confidence, upon opium in its capacity of procuring sleep,—or upon chloroform in the production of anæthesia. And I confidently believe that, in the future, it will be relied on with the same certainty and with as much confidence in its powers, as an arterial sedative, as we now do when we enter upon the exhibition of either of the above in those cases demanding their exhibition. Indeed, I am inclined to the opinion that there is much more certainty in its therapeautic action than in a majority of the drugs now used. Its diaphoretic powers were very manifest, and its expectorant influences were observed by Prof. Allen as well as myself, whose confidence in the *Veratrum* is equally strong with my own.

I feel that I have in part made the *amende* for the neglect with which I treated the suggestions, heretofore and repeatedly, presented touching the employment of this valuable remedy, and would recommend it to my medical friends as an agent of no ordinary properties and powers.

April 10, 1857.

